# Exploring boundary conditions of the single-code/default strategy in pigeons

**DOI:** 10.3758/s13420-024-00629-0

**Published:** 2024-04-26

**Authors:** Carlos Pinto, João Queiroz

**Affiliations:** School of Psychology, University of Minho, Gualtar Campus, 4710-057 Braga, Portugal

**Keywords:** Visual discrimination, Coding, Matching to sample, Many-to-one matching, Pigeon

## Abstract

To investigate the extent of adoption of more efficient coding strategies, pigeons learned, in three experiments, a symbolic matching-to-sample task that featured an asymmetric sample-comparison mapping. In all experiments, one comparison was correct following one of the samples (one-to-one mapping), and another comparison was correct following the remaining samples (many-to-one mapping). The experiments differed in sample number; Experiment 1 featured three samples, Experiment 2 five samples, and Experiment 3 seven samples. Our goal was to assess the adoption of a single-code/default coding strategy, which establishes two response rules: one rule specific to the sample mapped one-to-one (the single code), and another rule to be applied following any other sample (the default rule). Alternatively, the animals could establish more response rules, one per sample. Thus, the single-code/default strategy allows learning a task via a reduced number of response rules, and the more samples are mapped many-to-one, the greater the savings it allows. As such, the three experiments should progressively be more amenable to the adoption of this strategy. Overall, the adoption of a single-code/default strategy was not widespread. When taken together with previous results, the present study suggests that the amount of training may affect the coding strategy pigeons adopt. Additionally, our results underscore that individual differences are a fundamental aspect to consider when studying learning flexibility.

## Introduction

By retrieving information from its environment, an animal can establish response rules (or codes) that will subsequently regulate its behavior. Flexibility in these response rules is instrumental for finding solutions to the challenges posed by an ever-changing environment. Among these solutions, some are likely to be more adequate (i.e., yielding better results) or more efficient (i.e., more economical in the resources required) than others.

The establishment of response rules (known as coding) as well as flexibility in selecting the best solutions for a problem have been frequently studied via matching-to-sample tasks. In these tasks, following the presentation of a sample stimulus, there is a choice between two or more comparison stimuli. Choice of a specific comparison will be correct depending on the sample presented previously: for instance, in a task with samples S1 and S2 and comparisons C1 and C2, if S1 was presented C1 should be selected, and if S2 was presented C2 should be selected. In this case, learning the task could involve learning two sample-specific response rules: “If S1, choose C1” and “If S2, choose C2.” Alternatively, the animals could have adopted a “single-code/default” strategy, establishing one sample-specific code (e.g., “If S1, choose C1”), and a nonspecific, default code for all other samples (e.g., “If not S1, choose C2”). Following this strategy, C2 would always be chosen, unless information related to S1 was available at the moment of choice – C1 would be chosen in that case.

Based on the assumption that coding strategies depend on the particularities of each task, the adoption of a single-code/default strategy has been studied on a variety of matching tasks. For instance, when samples differ in salience, pigeons seem to establish a specific code only to the most salient sample (Grant, 2009; Wixted & Gaitan, 2004). Similarly, when amount of training is varied between samples, it appears that a specific code is created only for the sample that was trained more extensively (Grant, 2006; Grant & Blatz, 2004). Of most interest to the present work is a matching task that employs many-to-one mapping: one comparison (C1) is correct following two or more samples (e.g., S1, S2). By contrast, the other comparison (C2) is correct following only one sample (S3). In this case, instead of establishing three separate codes, one for each sample, a single-code/default strategy based on two codes – “If S3, choose C2” (single code) and “If not S3, choose C1” (default) – may be more efficient.

A method commonly used to study whether many-to-one tasks promote single-code/default coding is, after training the task, introducing a retention interval between sample offset and comparison onset. The rationale is that accurate performance requires the retention of information related to the sample (or to the comparison to be chosen) throughout the retention interval. The longer the retention interval, the more likely is the retained information to be lost. If an animal is using a single-code/default strategy, when faced with a choice following the loss of information, it should resort to the “default” response (since there is no information available regarding the sample for which the “single code” was established). Therefore, if an animal has adopted a single-code/default strategy, following long-enough retention intervals there should be a preponderance of choices to the “default” comparison, the comparison with the many-to-one mapping.

Gaitan and Wixted (2000) trained pigeons on two tasks where three samples, lasting 0, 2, and 10 s, mapped onto two colored comparisons: in one task, 0-s and 2-s samples shared their correct comparison (Experiment 2); in other task, 0-s and 10-s samples shared a comparison (Experiment 3). The results of subsequent retention tests were consistent with single-code/default coding: In both experiments, there was a preference for the comparison with the many-to-one mapping, what would be the “default” comparison. However, this evidence was not definitive. The interval that separated trials was spent in darkness, so in trials with 0-s samples, the pigeons could have learned to choose one comparison following a period of darkness. Since the retention interval was also spent in darkness, the results of the retention tests could be due to the birds selecting the comparison that, in training, was correct immediately following a period of darkness. In both experiments, the 0-s sample was part of the many-to-one mapping, so a preference for the 0-s comparison and the predictions of the single-code/default strategy were confounded.

To assess whether the similitude between inter-trial and retention intervals was of significance, Zentall et al., (2004) replicated Gaitan and Wixted’s (2000) Experiment 2, one condition with inter-trial and retention intervals dark, and other condition with an illuminated inter-trial interval. When the intervals were similar, a preference for the comparison associated with 0- and 2-s was found; when the intervals were different there was no difference in preference between comparisons. These results suggest that the evidence for single-code/default obtained by Gaitan and Wixted (2000) could in fact be an artifact introduced by the use of 0-s samples.

To avoid potential confounds with 0-s samples, Singer et al., (2006, Experiment 2) trained pigeons with three non-zero sample durations (2, 8, and 32 s). In their task, 2- and 32-s samples shared a comparison, and retention testing showed a preference for the comparison associated with these two samples, the result expected if the animals used a single-code/default strategy. However, this result is also consistent with the establishment of a code for each sample and a preference for the shortest sample on retention testing, a common result in retention testing with duration samples, known as choose-short effect (e.g., Grant & Spetch, 1993; Kraemer, Mazmanian & Roberts, 1985; Pinto & Machado, 2011; Spetch & Wilkie, 1982, 1983).

To disentangle the choose-short effect from single-code/default coding, Pinto and Machado (2015) trained pigeons in a task with three non-zero sample durations (2, 6, and 18 s), with the 6- and 18-s samples sharing the correct comparison. In retention testing, there was no choose-short effect; a strong preference for the comparison associated with 6- and 18-s samples was found, a result consistent with the single-code/default strategy. It is worth noting that the mapping used could have induced an alternative coding strategy, consisting of the establishment of a threshold between 2 s and 6 s, and responding being based on whether the sample duration was below or above the threshold. Even though this would not be a case of single-code/default, it would still be an example of coding flexibility, where three sample-specific codes would be replaced by two codes (“If below threshold, choose C1” and “If above threshold, choose C2”).

The more samples used, the more advantageous the single-code/default strategy can be: In a task with three samples, if an animal adopts a single-code/default strategy (as opposed to sample-specific codes), it establishes two codes instead of three. In a task with, say, five samples (in which four share a correct comparison), the savings brought by a single-code/default strategy are more significant: two codes versus five. Therefore, the more samples used, the greater the incentive to form a common, “default” code. As discussed in the previous paragraphs, the evidence for a single-code/default strategy in temporal discriminations is still not entirely clear, but it is worth noting that the tasks employed have used the minimum number of samples (three), the situation in which the benefit of a single-code/default strategy is arguably the lowest.

**Fig. 1:**
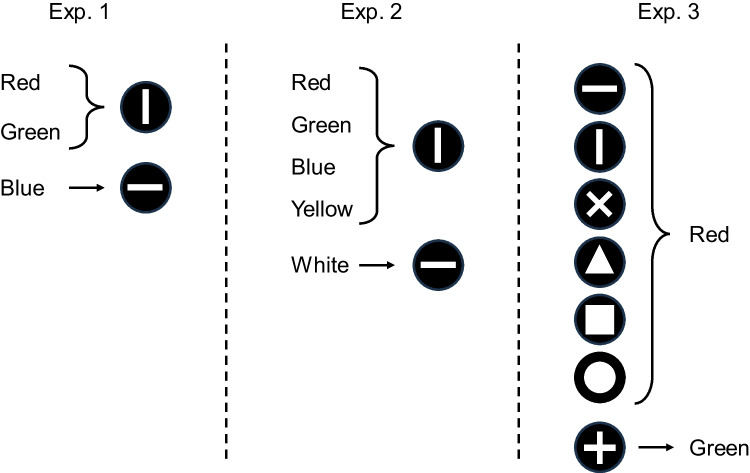
Schematic representation of the task in Experiment 1 (**left panel**), Experiment 2 (**center panel**), and Experiment 3 (**right panel**). In Experiments 1 and 2, following the presentation of a color, there was a choice between a vertical or a horizontal line. In Experiment 3, following the presentation of a shape, there was a choice between a red or a green stimulus. The diagrams show the correct choices. Sample-comparison mappings were counterbalanced in Experiments 1 and 2, one possible arrangement is depicted. In Experiment 3, the “plus” sign was always mapped one-to-one; the comparisons were counterbalanced

By contrast, on non-temporal discriminations, the task that has provided the strongest evidence of the adoption of a single-code/default strategy has employed five samples (Clement & Zentall, 2000), a procedure more favorable to the adoption of this coding strategy. In the present study, in three non-temporal discrimination tasks, we explored the conditions for the adoption of a single-code/default strategy. The first task was equivalent to those used with temporal discriminations, with three samples mapped onto two comparisons (Fig. 1, left panel). The second task was similar to Clement and Zentall (2000), and featured five samples (Fig. 1, center panel). Finally, the third task employed seven samples (Fig. 1, right panel). As the number of samples mapped onto a comparison increases, a single-code/default strategy becomes arguably more efficient, and we were interested in assessing whether the adoption of this strategy would become more prevalent.

## Experiment 1

Experiment 1 tested if a setup equivalent to the one used with temporal discriminations (three samples mapped onto two comparisons) would occasion the adoption of a single-code/default strategy in a non-temporal discrimination. Since this strategy is arguably more advantageous when more samples are mapped onto a comparison, a three-sample task is a boundary condition, given that the benefits of a single-code/default strategy over a sample-specific coding strategy are the smallest (two response rules vs. three, respectively).

### Method

#### Subjects

Eight pigeons (*Columba livia*) were maintained at approximately 80% of their free-feeding body weight. The room where the animals were kept was maintained in a 13:11 h light/dark cycle, with lights on at 08:00 and was kept between 20 ºC and 22 ºC. In each individual home cage water and grit were freely available. The experiment was conducted once a day, 7 days a week, at approximately the same time of day for each pigeon. The pigeons had no experience with color discrimination tasks, but all had experience with a matching-to-sample task that included many-to-one mapping (three samples, two comparisons), albeit featuring a temporal discrimination (Pinto & Machado, 2017).

#### Apparatus

Four experimental chambers were used (two birds were run in each chamber): Three LVE (Lehigh Valley Electronics) chambers and one homemade chamber. The LVE chambers were 34 cm high, 35 cm long, and 31 cm wide. One of the walls of the chamber featured three circular response keys, 2.5 cm in diameter, arranged horizontally. The three keys were 9 cm apart, center to center, and the bottom of each key was 22.5 cm above the wire mesh floor. Behind each key, a 12-stimulus IEE (Industrial Electronics Engineers) in-line projector presented visual stimuli. Colored stimuli were made by placing a piece of colored acetate sheet as a filter. Below the keys, a 6-cm wide, 5-cm high opening granted access to a food hopper with mixed grain. The hopper opening was centered horizontally, 8.5 cm above the floor, and was illuminated with a 28-V, 0.04-A light when the hopper was activated. On the opposite wall, 30 cm above the floor, a 28-V, 0.1-A houselight provided general illumination to the chamber. An exhaust fan circulated air and masked outside noises.

The homemade chamber had a similar setup to the LVE chambers. It measured 31 × 33 × 33 cm (h × l × w) and was equipped with three circular response keys, arranged horizontally. The keys, 2.5 cm in diameter and 21 cm above the wire mesh floor, were 9 cm apart (center to center). A 12-stimulus IEE (Industrial Electronics Engineers) in-line projector was installed behind each key. Access to a LVE food hopper was made through a 6-cm wide × 4.5-cm high horizontally centered opening, located below the response keys, 6.5 cm above the floor. When the hopper was activated, a 28-V, 0.04-A light illuminated its opening. On the opposite wall, 27.5 cm above the floor, a 28-V, 0.1-A houselight provided general illumination. The operant chamber was enclosed by a PVC sound-attenuating cubicle (Med Associates, ENV-018V) equipped with an exhaust fan.

The ABET II software (Lafayette Instrument Company) controlled experimental events and recorded the data.

#### Procedure

*Training.* A trial started with the sample stimulus (a red, green, blue, or white hue) presented on the center key. Following five pecks on the center key, the sample was removed and the two side keys were illuminated with the comparison stimuli, a horizontal and a vertical bar (both white on a black background). One response on a side key turned off both keys and, if the response was correct, was followed by access to food and a 30-s intertrial interval (ITI), during which the houselight illuminated the box. An incorrect response was followed immediately by the ITI. When the ITI ended, the houselight was turned off and a new trial started. A correction procedure was used, in which a trial was repeated following an incorrect response. In the trial following three consecutive incorrect responses, only the correct comparison was presented. Each animal saw three different sample colors. One comparison was correct following two samples (S1 and S2), while the other comparison was correct following one sample (S3). The stimuli corresponding to each sample and comparison were counterbalanced across pigeons.

Excluding correction trials, a session comprised 64 trials: 16 × S1 trials, 16 × S2 trials, and 32 × S3 trials. This trial proportion assured that, in a session, each comparison was correct the same number of times. Each comparison was presented the same number of times on each of the lateral keys. Training continued for a minimum of ten sessions, and until the animals were choosing the correct comparison following each sample at least 80% of the trials, for two consecutive sessions. To minimize feeding outside the session, reinforcement duration was adjusted individually, and varied from 2 s to 5 s across animals.

*Retention test*. On test trials, a retention interval was introduced between sample offset and comparison onset (a period of darkness that could last 2.5, 5, 10, or 20 s). A session comprised 80 trials: 48 regular training trials (12 × S1, 12 × S2, 24 × S3) and 32 retention-interval test trials (8 × S1, 8 × S2, 16 × S3), randomly interspersed. Each retention-interval duration was presented the same number of times following each sample. Table 1 details the structure of a session. In both training and retention-test trials, correct responses were always reinforced. The retention test lasted five sessions.

**Table 1:** Structure of a retention-test session in Experiment 1

Sample	Delay					Total
	0 s	2.5 s	5 s	10 s	20 s	
S1	12	2	2	2	2	20
S2	12	2	2	2	2	20
S3	24	4	4	4	4	40
Total	48	8	8	8	8	80

#### Data analysis

We analyzed choice behavior, namely matching accuracy, by the end of training and on retention tests. Statistical tests were conducted with the jamovi software for Windows (version 2.3.21), with Type-1 error rate set to 0.05. On retention tests, repeated-measures ANOVA compared performance following samples mapped to the same comparison (many-to-one samples), and also performance following many-to-one and one-to-one samples. As measure of effect size, we used the generalized eta square (${\upeta }_{G}^{2}$, e.g., Bakeman, 2005; Olejnik & Algina, 2003).

### Results

The pigeons required from 10 to 25 sessions ($\overline{x }=18$) to learn the task. By the last three sessions of training, average matching accuracy was 89.1% for the two samples mapped many-to-one (MTO) and 89.5 % for the sample mapped one-to-one (OTO).

**Fig. 2:**
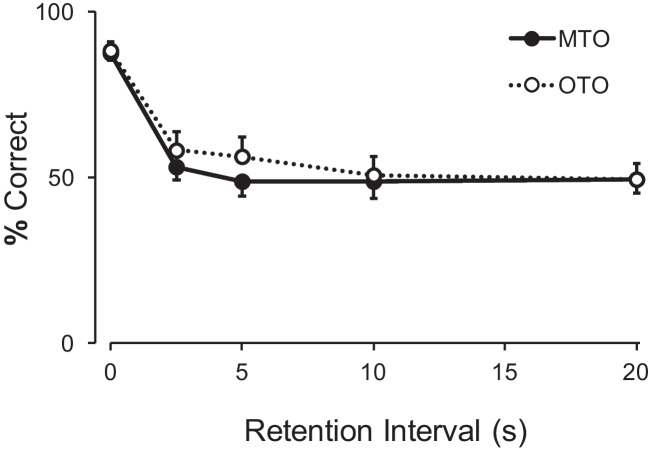
Mean (with SEM) percent correct to the two sample types (*MTO* many to one; *OTO* one to one) as a function of retention interval duration in Experiment 1

A repeated-measures ANOVA with sample (two levels) and retention interval (five levels) confirmed that performance following samples S1 and S2 (the samples sharing a comparison) did not differ; there was no significant main effect of sample, *F*(1, 7) = 0.97, *p* = .356, ${\eta }_{G}^{2}$ = 0.00, nor interaction, *F*(4, 28) = 0.88, *p* = .487, ${\eta }_{G}^{2}$ = 0.04. Since no significant difference was found between S1 and S2, they were combined in a single “many-to-one” category. To compare the performance of pigeons for the MTO and OTO samples, a repeated-measures ANOVA with sample (two levels) and retention interval duration (five levels) as factors revealed no significant main effect of sample, *F*(1, 7) = 0.20, *p* = .668, ${\eta }_{G}^{2}$ = 0.02, and a significant main effect of retention interval,* F*(4, 28) = 60.46, *p* < .001, ${\eta }_{G}^{2}$ = 0.59. The interaction was not significant, *F*(4, 28) = 0.48, *p* = .748, ${\eta }_{G}^{2}$ = 0.01. These results confirm that the retention interval had an effect on responding, but at the group level it did not differ between samples. Figure 2 shows the average performance in the retention test.

At the individual level, five of the eight pigeons were well described by the average function: the introduction of retention intervals led to a decrease in matching accuracy, with no clear preference for any comparison, and choice approached chance levels. One pigeon (PG45) preferred the MTO comparison, and two (P053, PG46) preferred the OTO comparison.

#### Discussion

In this experiment, pigeons learned a matching-to-sample task with three samples and two comparisons; one comparison was correct following two samples (many-to-one mapping) and the other was correct following the remaining sample (one-to-one mapping). This task has been commonly used to study the adoption of a single-code/default strategy in temporal discrimination tasks. The strategy consists in establishing two response rules, one specific to the sample mapped one-to-one, and a general, default rule applied to any other sample. A single-code/default strategy may be advantageous because the establishment of a response rule that applies to more than one sample should be more economical than establishing one response rule per sample.

The more samples share a correct comparison, the greater the savings brought by the adoption of a general response rule. Therefore, the three-sample task is the case in which the benefits of this strategy are smallest. When only two samples share a comparison, the adoption of a general rule reduces only one rule (going from two sample-specific rules to one general rule that would apply to both samples). The purpose of Experiment 1 was then to assess whether the three-sample version of the task would be sufficient to lead to the adoption of a single-code/default strategy in a non-temporal discrimination.

The average results of the retention test were not consistent with the single-code/default strategy: overall, all samples were similarly affected by the retention intervals. That is, a “default” response rule was seemingly not established. Therefore, it seems that the three-sample task was insufficient for the arguably more efficient single-code/default strategy to be widely adopted.

If a single-code/default strategy entails the establishment of fewer responses, it is possible that acquisition would be faster. In fact, the one pigeon that showed a preference pattern consistent with single-code/default strategy (PG45) was fastest to learn the task, taking only ten sessions to meet criterion. To illustrate, the second-fastest bird took 16 sessions to complete training.

## Experiment 2

In Experiment 1, a three-sample task was insufficient for the widespread adoption of a single-code/default strategy. To further our exploration of the boundary conditions of this strategy, in Experiment 2 two additional samples were added, so that now one comparison was mapped to four samples. That means that, if an animal adopts a single-code/default strategy (as opposed to sample-specific codes), it would establish two codes instead of five. Therefore, a single-code/default strategy may appear more appealing in Experiment 2, and thus be more widely adopted than in Experiment 1.

### Method

#### Subjects

Eight pigeons (*Columba livia*) participated in the experiment and were kept in the same living conditions as in Experiment 1. The pigeons had no experience with color discrimination tasks, and only one (P785) had experience with many-to-one mapping (three samples, two comparisons), but in a temporal discrimination (Pinto & Machado, 2015).

#### Apparatus

The apparatus was the same as in Experiment 1.

#### Procedure

##### **Training**

A session was similar to Experiment 1. Excluding correction trials, a session consisted of 80 trials, 40 many-to-one trials (with samples S1–S4 appearing ten times) and 40 one-to-one (S5) trials. As in Experiment 1, trial proportions were selected to equate how often each comparison was correct. Training continued for a minimum of 15 sessions and until matching accuracy to each sample was at least 80%, for two consecutive sessions, or until 30 sessions were completed. Reinforcement durations were adjusted individually and ranged from 1.25 to 4 s.

##### **Retention test**

Test sessions were similar to Experiment 1 but comprised 128 trials: 64 regular training trials – 32 × samples S1–S4 (each presented eight times) and 32 × sample S5 – and 64 retention-interval test trials – 32 × samples S1–S4 and 32 × sample S5. Please refer to Table 2 for the detailed structure of the session. Correct responses were reinforced in both training and retention-test trials. The retention test lasted five sessions.

**Table 2:** Structure of a retention-test session in Experiment 2

Sample	Delay					Total
	0 s	2.5 s	5 s	10 s	20 s	
S1	8	2	2	2	2	16
S2	8	2	2	2	2	16
S3	8	2	2	2	2	16
S4	8	2	2	2	2	16
S5	32	8	8	8	8	64
Total	64	16	16	16	16	128

#### Data analysis

The analyses run were the same as in Experiment 1.

## Results

Pigeons required from 15 to 30 sessions to learn the task (*x̅* = 20.3). One pigeon failed to learn the discrimination by session 30 and did not advance to testing. For the remaining pigeons, by the last three sessions of training, average matching accuracy for the four samples mapped many-to-one (MTO) was 93.1% (range: 91.4–95.7) and for the sample mapped one-to-one (OTO) was 88.8%.

**Fig. 3:**
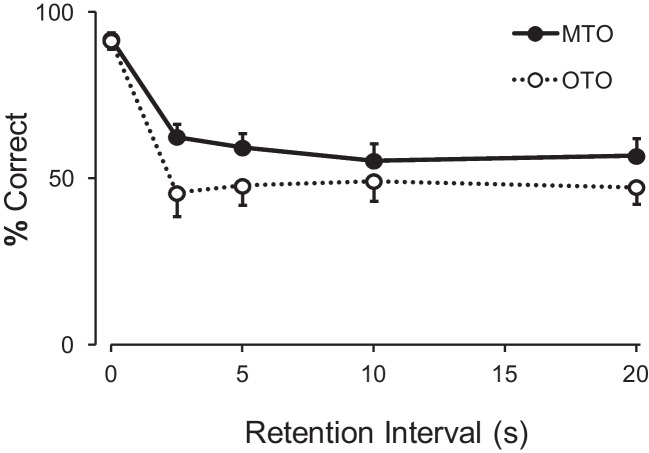
Mean (with SEM) percent correct to the two sample types (*MTO* many to one; *OTO* one to one) as a function of retention interval duration in Experiment 2.

A repeated-measures ANOVA with samples mapped many-to-one (four levels) and retention interval duration (five levels) as factors revealed no significant difference between samples, with no significant main effect of sample, *F*(3, 18) = 2.65, *p* = .08, ${\eta }_{G}^{2}$= 0.03 nor interaction, *F*(12, 72) = 1.29, *p* = .244, ${\eta }_{G}^{2}$ = 0.09. Since these four samples did not differ, they were grouped as MTO samples and compared with the OTO sample, S5. Figure 3 shows the average matching accuracy following MTO and OTO samples on the retention test.

A repeated-measures ANOVA with sample type (two levels) and retention interval duration (five levels) as factors revealed a significant main effect of retention interval, *F*(4, 24) = 96.40, *p* < .001, ${\eta }_{G}^{2}$ = 0.62, but no significant main effect sample type, *F*(1, 6) = 1.43, *p* = .276, ${\eta }_{G}^{2}$ = 0.12. The interaction was also not significant, *F*(4, 24) = 1.14, *p* = .362, ${\eta }_{G}^{2}$ = 0.05. These results show that the overall effect of the retention intervals did not differ between samples.

At the individual level, five of the seven pigeons followed the pattern described by the average function, with accuracy following MTO and OTO samples approaching indifference. One pigeon, P746, showed a preference for the MTO comparison, and another, P785, showed a preference for the OTO comparison.

## Discussion

In Experiment 2, the number of samples was increased so that now one comparison was correct following four samples. This sample-comparison mapping could have been more conducive to the adoption of a single-code/default strategy, but overall results were similar to Experiment 1. While in Experiment 1 the group data showed the MTO and OTO functions overlapping (Fig. 2), in Experiment 2 accuracy following MTO samples was consistently, albeit slightly, above accuracy for OTO samples (Fig. 3). However, the difference between sample type was not significant, so, while suggestive of the pattern expected were a single-code/default strategy adopted, the results do not allow us to conclude that the pigeons were following such a strategy. The individual data corroborate this reading: for most pigeons accuracy in general approached indifference. In conclusion, the increase in sample number from Experiment 1 to Experiment 2 did not result in a stronger adoption of a single-code/default strategy.

Experiment 1 suggested a correlation between coding strategy and speed of acquisition, which was not confirmed in Experiment 2. Contrary to Experiment 1, the pigeon that showed a preference for the “many-to-one” comparison in retention testing was not the fastest to acquire the discrimination; in fact, was one of the slowest (27 sessions). The pigeons that reached 80% accuracy to all samples the fastest (eight sessions) were indifferent between comparisons in the retention test.

The sample-comparison mapping in this experiment was similar to Clement and Zentall (2000), a study that found evidence consistent with an overall adoption of a single-code/default strategy. In the present experiments there has been some individual variability in the retention test, so the difference between Clement and Zentall (2000) and Experiment 2 could be due to sampling effects. However, there is another difference of note between the studies: the amount of training. While in the present experiment pigeons were tested following an average of 20.3 sessions of training, in Clement and Zentall (2000) training lasted an average of 177.6 sessions in one experiment, and of 82.0 sessions in another. Sessions were also slightly longer in Clement and Zentall (2000): 96 trials versus 80 trials in the current experiment. Amount of training can affect performance, such as in memory (e.g., D'Amato, 1973; Grant, 1976) or learning-set tasks (e.g., Warren, 1965), so coding strategies may be similarly susceptible, and a widespread adoption of a single-code/default strategy may require a large number of training sessions.

## Experiment 3

Experiments 1 and 2 did not reveal a widespread adoption of a single-code/default strategy. This could be because this strategy, even though requiring fewer response rules than a sample-specific coding strategy, may be more difficult to learn. As such, it may require more time to learn, or it will be adopted only under conditions where its benefits are significant. Thus, in Experiment 3 we added two more samples, so that the training task now employed seven samples (six of them mapped to the same comparison). If a pigeon adopts a single-code/default strategy (instead of establishing sample-specific codes), it would need to learn only two codes instead of seven, a significant difference.

Given that this experiment is the one most likely to predispose the adoption of the single-code/default strategy, two more tests were included. In one test a new sample was presented; if the pigeons developed a default code, when faced with an untrained sample they should prefer the “default” comparison. In the other test, no sample was presented; we were interested in seeing if, in the absence of any sample, there would be a preponderance of “default” choices.

### Method

#### Subjects

Eight pigeons (*Columba livia*) participated in the experiment and were kept in the same living conditions as in the previous two experiments. No pigeon had experience with color discrimination tasks. Half of the pigeons (P192, P452, P902, P9131) had participated in an experiment that featured many-to-one mapping (three samples, two comparisons), in a temporal discrimination (Pinto & Machado, 2023).

#### Apparatus

The apparatus was the same as in the previous experiments.

#### Procedure

##### Training

Sessions were similar to Experiments 1 and 2, with the difference that training employed seven samples (a vertical bar, a horizontal bar, a circle, a square, a triangle, an “x” mark, and a “plus” sign). All samples were white on a black background. Shapes were used as samples in this experiment due to limitations on the number of colors available in the operant chambers. A red and a green hue were used as comparison stimuli. Six samples (S1–S6) were mapped to one comparison and the remaining sample (S7) was mapped to the other comparison. For all pigeons, the sample mapped one-to-one (S7) was the “plus” sign. Given the similarity between the “plus” sign and several other stimuli (the vertical and horizontal bars, and the “x” mark), if they shared the same comparison, they could be treated as a single sample (e.g., “sample with a vertical line down the middle” or “sample with two intersecting lines”). In that event, the planned six-to-one sample-comparison mapping would effectively be a five-to-one mapping. While it is not possible to ensure what stimulus characteristic an animal attends to, we opted for this arrangement given that the “plus” sign was arguably the stimulus that shared similarities with most of the other stimuli.

Not considering correction trials, a session consisted of 120 trials: 60 many-to-one trials (samples S1–S6 were presented ten times each) and 60 one-to-one (S7) trials. Training lasted a minimum of 15 sessions and a maximum of 35 sessions, and persisted until the pigeons were selecting the correct comparison after each sample in at least 80% of the trials, for three consecutive sessions. Reinforcement durations varied from 1 s to 3 s across animals.

##### Retention test

The test was similar to the previous experiments; a session consisted of 120 trials, 72 regular training trials (with no retention interval), and 48 retention-test trials. Regarding the regular training trials, there were 36 presentations of samples S1–S6 (each presented six times), and 36 presentations of sample S7. Regarding the retention-test trials, samples S1–S6 were presented four times each (once per retention interval), and sample S7 was presented 24 times (six times per retention interval). Please refer to Table 3 for an overview of the structure of the session. Half of the retention-test trials were reinforced, non-differentially. Given that each session contained few test trials per sample, this test was run for ten sessions, twice as long as in the previous two experiments.

**Table 3:** Structure of a retention-test session in Experiment 3

Sample	Delay					Total
	0 s	2.5 s	5 s	10 s	20 s	
S1	6	1	1	1	1	10
S2	6	1	1	1	1	10
S3	6	1	1	1	1	10
S4	6	1	1	1	1	10
S5	6	1	1	1	1	10
S6	6	1	1	1	1	10
S7	36	6	6	6	6	60
Total	72	12	12	12	12	120

##### Retraining

Pigeons returned to Training for a minimum of three sessions, and moved to the next test when a three-session average accuracy to each sample was at least 70%.

##### New-sample and no-sample test

Afterwards, the pigeons went through five sessions of the New-sample and no-sample test. A session comprised 116 trials, 96 regular training trials and 20 test trials. Regular trials were divided in 48 trials with samples S1–S6 (each presented eight times) and 48 trials with sample S7. In half of the test trials, a new sample (white hue) was presented, and then the trial proceeded as a regular trial. In the remaining test trials, no sample was presented, so a trial began with the presentation of the two comparisons. In new- and no-sample tests, half of the trials ended with reinforcement, delivered non-differentially.

#### Data analysis

In addition to the same analyses as in Experiments 1 and 2, to compare choices of the “many-to-one” comparison with chance levels in delayed, new-sample, and no-sample trials, we calculated 95% confidence intervals (normal approximation to the binomial) for each pigeon. Choices on retention-test trials (all delays combined) were compared with choices on new-sample trials via a Pearson’s correlation (Kim, 2015).

### Results

Pigeons required from 18 to 35 sessions to learn the task (*x̅* = 26.9). By the 35th session, one pigeon still had not learned the correct response to one of the samples, and so did not proceed to testing. For the remaining pigeons, by the last three sessions of training, average matching accuracy for the six samples mapped many-to-one (MTO) was 92.9% (range: 88.6–97.1) and for the sample mapped one-to-one (OTO) was 91.7%. In the first three sessions of retraining, average accuracy for the six MTO samples was 94.2% (range: 88.1–97.6) and for the OTO sample was 93.7%. All pigeons progressed to the next test after those three sessions of retraining.

**Fig. 4:**
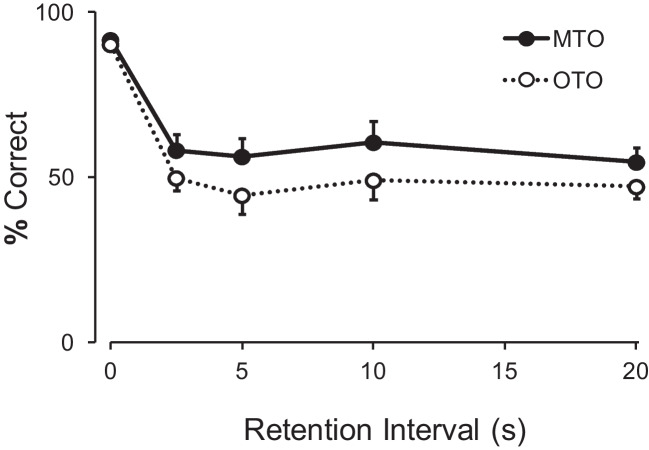
Mean (with SEM) percent correct to the two sample types (MTO many to one; OTO one to one) as a function of retention interval duration in Experiment 3

A repeated-measures ANOVA with samples that shared a comparison (six levels) and retention interval duration (five levels) as factors revealed no significant difference between these samples: there was no significant main effect of sample, *F*(5, 30) = 1.37, *p* = .264, ${\eta }_{G}^{2}$ = 0.01 or interaction, *F*(20, 120) = 0.87, *p* = .629, ${\eta }_{G}^{2}$ = 0.05. Therefore, these samples were grouped into a single “many-to-one” category for the following analyses. Figure 4 illustrates the average performance in the retention test for MTO and OTO samples. 

To compare delayed choices following MTO and OTO samples, we ran a repeated-measures ANOVA with sample (two levels) and retention interval duration (five levels) as levels. There was a significant main effect of retention interval, *F*(4, 24) = 124.41, *p* < .001, ${\eta }_{G}^{2}$ = 0.65, but no significant main effect of sample, *F*(1, 6) = 1.40, *p* = .281, ${\eta }_{G}^{2}$ = 0.11 or of interaction, *F*(4, 24) = 0.56, *p* = .691,${\eta }_{G}^{2}$ = .003. That is, overall there was no preference for either sample type following a delay.

At the individual level, for three pigeons choices were around chance levels in trials with a retention interval (95% confidence interval (CI) for choices of the “many-to-one” comparison included 50%). Three pigeons preferred the MTO comparison, 95% CI [64.6%, 72.9%], [51.8%, 60.7%], and [63.1%, 71.5%]. Finally, one pigeon preferred the OTO comparison, 95% CI [30.7%, 39.3%].

**Fig. 5:**
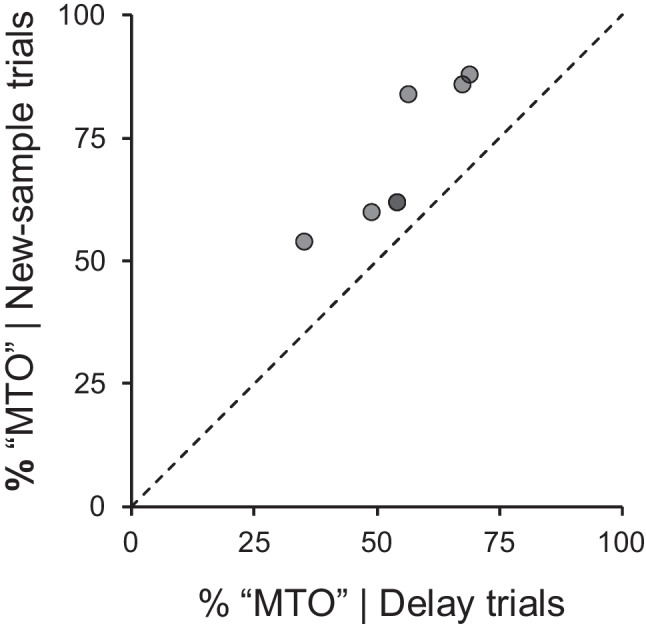
Percent of choices of the “many-to-one” comparison in delay test trials (x axis) plotted against percent of choices of the “many-to-one” comparison in new-sample test trials (y axis). Each data point refers to one pigeon. The dashed line is the line of equality

In no-sample trials, the MTO comparison was chosen on 46.9% of the trials, a preference that did not differ significantly from chance, 95% CI [41.6%, 52.1%]. When a new sample was presented, there was an overall preference for the MTO comparison, chosen on 70.9% of the trials, a preference significantly different from chance, 95% CI [66.1%, 75.6%]. A pigeon’s choices on retention-test trials (all delays combined) correlated positively with choices on new-sample trials, *r*(5)* =* .87, *p =* .011. Figure 5 contrasts, for each pigeon, choices of the MTO comparison on delay and new-sample trials.

### Discussion

In Experiment 3, the number of samples was once more increased; the pigeons learned a task with seven samples, six of them associated with the same comparison. Of all our tasks, this was arguably the most favorable to the adoption of a single-code/default strategy, not only by the savings brought by the adoption of a “default” response rule, but also because the establishment of seven sample-specific response rules would be onerous.

However, overall retention functions were similar to Experiment 2, with accuracy following the “many-to-one” samples slightly above the “one-to-one” sample, albeit non-significantly (see Figs. 3 and 4). Despite the similarities at the group level, this experiment was the one with the most pigeons showing a preference for the “many-to-one” comparison, the outcome expected if a “default” rule had been created. Even so, this was true for only three out of the seven pigeons. That is, even though it was in this experiment that the adoption of the single-code/default strategy was most evident, the majority of pigeons did not show it.

The new-sample test corroborated the results of the retention test; the three pigeons that preferred the “many-to-one” comparison in retention testing were the ones who chose that comparison the most when faced with a new sample, choosing it on 86% of the trials. Conversely, the pigeon that preferred the “one-to-one” comparison in retention testing was the one that chose the “many-to-one” comparison the least in the new-sample test (54% of the trials). For the remaining pigeons, the “many-to-one” comparison was chosen on approximately 61% of the new-sample trials. In the no-sample test, most pigeons’ preferences were around chance levels. Taking together the three tests, the retention and new-sample tests suggest that a subset of the pigeons adopted a single-code/default strategy; the results of the no-sample test show that the absence of a sample was not sufficient to trigger their “default” response.

Finally, Experiment 3 reinforced the conclusion brought by Experiment 2 that speed of acquisition is not related to performance in the retention test. Among the three pigeons that preferred the “many-to-one” comparison in the retention test, there were both the slowest and fastest to acquire the discrimination. There does not seem to be evidence that the establishment of fewer response rules results in speedier learning of a task.

## General discussion

In a series of three experiments, pigeons learned a symbolic matching-to-sample task where one comparison was associated with one sample and the other comparison was associated with multiple samples. The number of samples sharing a comparison was progressively increased: two in Experiment 1, four in Experiment 2, and six in Experiment 3, to assess to what extent a single-code/default coding strategy would be adopted. This strategy consists of the establishment of two response rules: one rule specific to the sample mapped one-to-one, and a default rule for all other cases. Given that it consists of only two rules, it is arguably more efficient than a strategy that establishes one specific rule per sample. The more samples, the greater savings a single-code/default strategy provides, so our experiments were expected to progressively be more amenable to this strategy. However, most pigeons did not appear to adopt a single-code/default strategy. When this strategy is followed, a preference for the comparison mapped many-to-one (the “default” response) is expected in retention testing. In Experiment 1, accuracy following all samples was similar; in Experiments 2 and 3, accuracy following the “many-to-one” samples was higher than following the “one-to-one” sample, but only to a slight degree: approximately 10%.

The strongest evidence for the general adoption of the single-code/default strategy has been provided by Clement and Zentall (2000), in a task where four samples shared a comparison. In that study, the effect of retention testing was approximately twice as large: accuracy following the “many-to-one” samples was roughly 25% higher than following the “one-to-one” sample.^1^ Knowing how widespread this strategy was at the individual level would allow for a better comparison between studies, but as discussed in Experiment 2, this difference possibly reveals another boundary condition for coding strategies: the amount of training. In Clement and Zentall (2000), the pigeons had substantially more training than in the present studies, and that may have led to a more widespread adoption of a single-code/default strategy: for instance, it is possible that individual sample-comparison associations are established initially, and with overtraining a single-code/default strategy may develop. Overtraining has been found to affect performance in multiple ways; for instance, in identity and oddity matching overtraining has been found to facilitate the learning of new discriminations (e.g., Nakagawa, 1992, 2001). Also, as aforementioned, overtraining can improve accuracy on delay tests (e.g., D’Amato, 1973; Grant, 1976), or determine the success in developing learning sets (e.g., Warren, 1965).The effect of amount of training on adoption of coding strategies is certainly worth exploring further (such as by running probe delay and new-sample tests at different points during acquisition).

In order to not bias choices, the number of reinforcements associated with each comparison was equated. As a consequence, the sample mapped one-to-one was presented more frequently than the other samples (for instance, in Experiment 3 sample S7 was presented six times more often than any of the other samples). Sample frequency has been found to bias preference in retention testing (DiGian, & Zentall, 2007), an effect that could have weakened the expression of a “default” response rule in the present studies. On the one hand, the more the samples mapped many-to-one, the more efficient a “default” rule is and, assumedly, more likely to be adopted. Such a rule would result, in testing, in choices of the “many-to-one” comparison. On the other hand, to equate reinforcement following both types of sample, the more the samples mapped many-to-one, the more frequently the “one-to-one” sample is presented. In testing, this could result in choices of the “one-to-one” comparison. It is possible that this latter effect led some pigeons to show a preference for the “one-to-one” comparison in retention testing. It is worth noting that, even though Clement and Zentall (2000) featured a similar preponderance of the “one-to-one” sample, there was an overall preference for the “many-to-one” comparison. It would be interesting to see whether in that study there was also a subset of pigeons showing a preference for the “one-to-one” comparison (even if to a smaller degree, as their average data suggests). It may be possible that birds differ in how sensitive they are to the asymmetric distribution of samples, and in that case, differences between experiments could be the result of sample differences. Further replications could clarify the frequency in which a “one-to-one” preference occurs in this task.

In the three experiments, retention functions showed a steep decline, and approached indifference in all delays tested. These patterns are not uncommon when delays are introduced only in testing, (e.g., Grant & Talarico, 2004; Pinto & Machado, 2015, 2017; Pinto & Sousa, 2021; Sherburne et al., 1998; Spetch, 1987; Spetch & Rusak, 1992), and may reflect a strong generalization decrement effect. When delays are present since the beginning of training – and new delays are used in testing – retention functions tend to decrease more gradually (e.g. Dorrance et al., 2000; Kelly & Spetch, 2000; Spetch & Rusak, 1989). It may be interesting to replicate these studies with delays since beginning of training, to assess if (and how) performance would differ.

As illustrated in the *Introduction*, it can be challenging to find a task that allows a definite identification of the coding strategy animals may be following, and the example in the previous paragraph underscores the difficulty in designing an ideal test. As such, comparing various training and testing procedures is advantageous; for instance, in Experiment 3 the combination of retention and new-sample tests allowed identifying the pigeons following a single-code/default strategy with greater certainty. Also, the no-sample test showed that not all situations where a “single-code” sample is absent lead to a “default” response.

Animals vary in how they learn a task; for instance, depending on task difficulty, animals may adopt a prospective or a retrospective coding strategy (Zentall et al., 1989). Even when faced with the same task, different animals may sometimes attend to different stimuli or learn different things (e.g., Reynolds, 1961; Gaitan & Wixted, 2000; Pinto, Fortes, & Machado, 2017; Pinto & Machado, 2023). In the case of the present tasks, even though they allowed for a coding strategy that is, arguably, more economical, most pigeons did not appear to make use of it. Taking into account that different animals may approach tasks differently or be sensitive to different task characteristics, perhaps a more adequate research question is to ask how common a coding strategy is in a given task. That is, when looking at how animals solve a task, instead of a single common solution, it is perhaps more adequate to adopt a plural view, with different solutions, some more likely to be adopted than others. The more data collected on the conditions, boundaries, and dynamics of adoption of coding strategies, the better will be our understanding of how animals adapt to and solve the environmental challenges they face.

## Footnotes

1 Accuracy values estimated from figure.

## Declarations

### Open practices statement

We report all data exclusions (if any), all manipulations, and all measures in the study. All data are available at https://doi.org/10.34622/datarepositorium/VVW399. This study's design and analysis were not pre-registered.
